# Association of Nat2 Gene Polymorphism with Antitubercular Drug-induced Hepatotoxicity in the Eastern Uttar Pradesh Population

**DOI:** 10.7759/cureus.4425

**Published:** 2019-04-10

**Authors:** Divya Yadav, Rahul Kumar, Rakesh K Dixit, Surya Kant, Ajay Verma, Kanchan Srivastava, S K Singh, Sarvesh Singh

**Affiliations:** 1 Miscellaneous, King George’s Medical University, Lucknow, IND; 2 Miscellaneous, King George's Medical University, Lucknow, IND; 3 Respiratory Medicine, King George's Medical University, Lucknow, IND; 4 Community Medicine, King George's Medical University, Lucknow, IND; 5 Experimental Pharmacology, King George’s Medical University, Lucknow, IND

**Keywords:** antituberculosis drugs, genotype, hepatotoxicity, slow acetylator, n-acetyltransferase 2

## Abstract

Introduction

Tuberculosis (TB) remains an important cause of morbidity and mortality worldwide. There are more than 20 drugs available for TB treatment. Hepatotoxicity is the most serious adverse drug reaction of anti-TB drugs. Various pathogenesis and genetic factors are associated with antituberculosis drug-induced hepatotoxicity (ATDIH). Antituberculosis drugs (ATDs) are mostly metabolized by N-acetyltransferase 2 (NAT2). Therefore, in this study, we aim to evaluate the role of the NAT2 genotype in ATDIH in the eastern Uttar Pradesh population.

Methods

A total of 100 TB patients who had been treated with anti-TB drugs were enrolled in this studied. In this group, 70 TB patients did not develop drug-induced hepatotoxicity (tolerant control group) and 30 TB patients developed ATDIH (ATDIH group). The genetic polymorphisms of the NAT2 genes were analyzed by polymerase chain reaction-restriction fragment length polymorphism (PCR-RFLP). Genotype and allele frequencies were evaluated by the t-test and odds ratio (OR) with 95% confidence intervals (CIs) were used to evaluate the strength of the associations.

Results

There is a high percentage of slow acetylators in the Eastern Uttar Pradesh population. Four percent of people are fast acetylators, 34% are intermediate acetylators, and 62% are slow acetylators. The frequency of slow acetylators in the NAT2 genotype was commonly present and was not significantly different between the ATDIH (73.33%) and tolerant control groups (61.40%). However, the genotypic distribution of variants of slow-acetylator genotypes (NAT2*6/7, NAT2*5/7, and NAT2*5/6) was also not significantly different in ATDIH.

Conclusion

In the present study, the slow acetylators of the NAT2 genotype did not contribute to the elevated risk of ATDIH development in tuberculosis patients.

## Introduction

According to the Global Tuberculosis Report 2017, the incidence of tuberculosis (TB) in India was approximately 28,00,000, that is, one-fourth of the world’s TB cases [1]. The Government of India started the Revised National Tuberculosis Control Programme (RNTCP) in the year 1992, headed by an expert committee, for the entire country, in a phased manner. This program helps face the disease in a more managed and classified form by incorporating a new drug regimen - The Directly Observed Treatment Short Course (DOTS) [2]. DOTS makes available free and compulsory good-quality antitubercular drugs under direct supervision, making the program more effective. According to the Union Ministry of Health and Family Welfare, 2.6 lakh TB cases were reported in Uttar Pradesh in 2016.

A combination of five antituberculosis drugs - isoniazid (H), rifampicin (R), ethambutol (E), pyrazinamide (Z), and streptomycin (S) - was recommended as first-line treatment. But, the success rate of these treatments depends upon the pharmacokinetics of individuals. Hepatotoxicity is a serious adverse effect due to the use of antituberculosis drugs (ATDs) in great TB burden countries. The occurrence of ATD-induced hepatotoxicity affects the management of TB, leading to delays or the failure of the treatment [3-5].

ATDs are mostly metabolized by N-acetyltransferase 2 (NAT2). The inactive function of NAT2 against isoniazid (H) is because it is a slow acetylator and has a higher risk of developing ATD-induced hepatotoxicity [3-6]. NAT2 plays a key role in the detoxification and elimination of drugs, and it is also involved in carcinogen metabolism [7]. It has been observed that modifications in NAT2 activity could result in the accumulation of precursors such as hydrazine and acetylhydrazine, leading to the development of hepatotoxicity [8].

In the human NAT2 gene, various single-nucleotide polymorphisms (SNPs) have been identified, which are responsible for the observed phenotypes. NAT2 is a highly polymorphic enzyme, and several mutations affect its activity. These mutations result in three different phenotypes: rapid acetylators (RAs), intermediate acetylators (IAs), and slow acetylators (SAs) [9]. NAT2*4 is considered the reference allele in the absence of all the known SNPs and is designated as a rapid allele. NAT2*5, NAT2*6, and NAT2*7 are the major groups of alleles that are associated with decreased enzyme activity (due to amino acid changes) and they form the SA phenotype [10]. A heterozygous compound genotype (NAT2*4/*5, NAT2*4/*6, or NAT2*4/*7) is considered the intermediate acetylator. These data are used by pharmacogenetics for deciding the dose of isoniazid on the basis of phenotype and genotype, in order to avoid hepatotoxicity [11].

In Asian populations, some studies have indicated that an SA status was associated with INH-induced hepatic disorders [8]. Due to the wide variability in the results of these studies and others, there is still considerable debate over this finding. Indians are members of one of the most heterogeneous populations in the world, and the role of NAT2 gene polymorphisms in ATDs-induced hepatotoxicity has not yet been fully established in the north Indian population who are at a higher risk of hepatotoxicity. Therefore, elucidating the genetics involved in anti-TB drug-induced hepatotoxicity in the Eastern Uttar Pradesh population would be of clinical significance and may provide valuable information to the medical community before starting ATDs. In this study, we will examine cases of H, R, and Z-induced hepatotoxicity (ATDIH) in patients taking ATD in order to elucidate the possible influence of genetic polymorphisms of NAT2 on the prevalence of hepatotoxicity. Moreover, we will also explore the distribution of NAT2 gene polymorphism (NAT2*4, *5, *6, and *7) in TB patients of Eastern Uttar Pradesh.

## Materials and methods

In this prospective study, patients of age more than 18 years were recruited from the outpatient department or the inpatient facility of the departments of pharmacology and therapeutics, clinical hematology, and respiratory medicine, Gandhi Memorial and Associated Hospitals, King George’s Medical University, Lucknow, Uttar Pradesh, India, between November 2017 and September 2018 after obtaining ethical clearance from the institute’s ethical committee.

A total of 100 TB positive patients were recruited in this study. Further, the TB patients were divided into two groups on the basis of anti-TB drugs (ATDs)-induced toxicity. After treatment with ATDs, 30 pulmonary TB patients developed clinical and/ or laboratory-confirmed DIH, whereas 70 patients with pulmonary TB did not develop DIH and constituted the controls (tolerant control group). Informed written consent was obtained from all TB patients. Clinical data, such as disease stage, liver function test, treatment details, and a history of comorbid conditions, were extracted from the patients’ charts.

Blood collection and molecular analysis

Blood samples (3 ml) were collected in ethylenediaminetetraacetic acid (EDTA) tubes from TB patients and stored at −80 °C until deoxyribonucleic acid (DNA) extraction. The genomic DNA was extracted from the blood specimens using Qiagen Blood DNA Mini Kit (Qiagen, Hilden, Germany) according to the manufacturers’ instructions.

N-acetyltransferase 2 (NAT2) genotyping

Genotyping of NAT2 polymorphism was performed by using polymerase chain reaction-restriction fragment length polymorphism (PCR-RFLP). The PCR was performed in a total 50 μl reaction volume containing 0.5 mM MgCl2, Taq polymerase buffer, 0.2 mM dNTPs, 10 pmoles each of both forward primer and reverse primer, and 1 unit of Taq DNA polymerase. Initial denaturation at 94 °C was performed for 5 min, followed by 30 cycles of denaturation at 94 °C for 1 min, annealing at 55 °C for 1 min, the elongation step at 72 °C for 1 min, and a final cycle of elongation at 72 °C for 7 min. The primer sequences for NAT2 were 5′ATGGACATTGAAGCATATTTT GAA AGAATT3′ (forward) and 5′AAGGGTTTATTTTGTTCCTTATTCTAAAT3′ (reverse).

The PCR product of the NAT2 gene (895 bp) was digested with the KpnI, TaqI, and BamHI restricted enzymes. The digested products were resolved on a 3% agarose gel. The loss of restriction cutting sites was represented as a NAT2*4 wild-type allele, whereas digestion with KpnI, TaqI, and BamHI was the NAT2*5, NAT2*6, and NAT2*7 allele, respectively. The obtained fragment sizes of allele were analyzed on a 3% agarose gel.

Statistical analysis

Data analysis was performed using SPSS version 21.0 (SPSS Inc., Chicago, Illinois, USA). Data were expressed as mean, median, and percentage. The genotypic distribution was compared between cases and controls using the t-test. The odds ratio (OR) of ATDIH associated with the NAT2 genotype was calculated using logistic regression. p<0.05 was considered statistically significant.

## Results

The demographic profiles of TB patients of the ATDIH group and the tolerant control group are shown in Table 1. The demographic profile includes age (32.20±7.41 years and 33.38±9.75 years), gender (male: female (60%:40% and 51.5%:48.5%), height (162.70±8.84 cm and 161.24±7.64 cm), weight (18.09±2.65 kg and 49.97±10.63 kg), body mass index (BMI) (18.09±2.65 kg/m^2^ and 49.97±10.63 kg/m^2^), aspartate aminotransferase (AST, 25.47±6.28 and 26.89±7.23), and alanine aminotransferase (ALT, 24.89±5.89 and 24.07±7.13) in the ATDIH group and tolerant control group, respectively. There was no significant difference in demographic profile between the ATDIH and tolerant control groups at the baseline. After one month of treatment, AST was 139.60±72.70 and 27.61±16.46 and ALT was 101.47±52.55 and 33.43±23.49 in the ATDIH group and tolerant control group, respectively. There was a significant increase in AST and ALT in ATDIH as compared to the tolerant control group after one month of treatment (Table 1).

**Table 1:** Demographic profile of patients in the ATDIH and tolerant control groups Data are represented as mean, ±SD. SD: standard deviation, ATDIH: antituberculosis drug-induced hepatotoxicity; BMI: body mass index; AST: aspartate aminotransferase; ALT: alanine aminotransferase; LFT: liver function test ^1^Unpaired t-test, *=Significant (<0.05)

	ATDIH Group (n=30)	Tolerant Control Group (n = 70)	^1^p-value
Age (years) Mean±SD	32.20±7.41	33.38±9.75	0.669
Gender			
Female (%)	12 (40.0%)	34 (48.5%)	0.224
Male (%)	18 (60.0%)	36 (51.4%)	
Height (cm)	162.70±8.84	161.24±7.64	0.407
Weight (kg)	47.83±7.80	49.97±10.63	0.324
BMI (kg/m^2^)	18.09±2.65	19.10±3.12	0.128
Baseline LFT			
AST	25.47±6.28	26.89±7.23	0.728
ALT	24.89±5.89	24.07±7.13	0.885
LFT at 1 month of treatment			
AST	139.60±72.70	27.61±16.46	<0.001^*^
ALT	101.47±52.55	33.43±23.49	<0.001^*^

In the ATDIH group, the localization of TB patients was pulmonary (76.67%), extrapulmonary (3.33%), and both pulmonary as well as extrapulmonary (20.0%). In the tolerant control group, the localization was 78.57% pulmonary only, 2.89% extrapulmonary, and 18.57% both pulmonary as well as extrapulmonary   (Table 2). The distribution of TB patients based on treatment type is shown in Table 2. The distribution of subjects was: 21 (70.0%) and 45 (64.29%) were taking Category 1 treatment and nine (30.0%) and 25 (35.71%) were taking Category 2 treatment in the ATDIH group and the tolerant control group, respectively. A total of nine (30.0%) and six (8.57%) smokers and five (16.67%) and three (4.29%) drug abusers were present in the ATDIH and tolerant control groups, respectively. A comparison of smokers, alcoholics, and drug abusers between groups is shown in Table 2.

**Table 2:** Distribution of TB patients on smokers, alcoholics, and drug abusers Data are represented as n (%), TB: tuberculosis; ATDIH: antituberculosis drug-induced hepatotoxicity ^1^Unpaired t-test

	ATDIH Group (n=30)		Tolerant Control Group (n = 70)	^1^p-value
Localization				
Pulmonary	23 (76.67%)		55 (78.57%)	0.976
Extra pulmonary	1 (3.33%)		2 (2.89%)	
Both	6 (20.0%)		13 (18.57%)	
Treatment type				
Category 1		21 (70.0%)	45 (64.29%)	0.65
Category 2		9 (30.0%)	25 (35.71%)	
Smokers (%)	9 (30.0%)		6 (8.57%)	0.571
Drug abusers (%)	5 (16.67%)		3 (4.29%)	0.71

In the ATDIH group, the frequency of fast acetylators was (0%), intermediate acetylators was (36.67%), and slow acetylators was (73.33%). In the tolerant control group, the frequency was: fast acetylators (5.71%), intermediate acetylators (33.85%), and slow acetylators (61.40%). There was no significant difference between the ATDIH group and the tolerant control group (Table 3).

**Table 3:** Distribution of patients on the basis of N-acetyltransferase-2 (NAT2) genotypes in the ATDIH and tolerant control groups Data are represented as n (%). ATDIH: antituberculosis drug-induced hepatotoxicity; OR: odds ratio; CI: confidence intervals

Acetylator status	ATDIH group (n=30)	Tolerant control group (n=70)	OR (CI)	p-value
Fast acetylators	0 (0%)	4 (5.71%)	Reference	-
Intermediate acetylators	11 (36.66%)	23 (32.85%)	0.22 (0.01-4.59)	0.4432
Slow acetylators	19 (63.33%)	43 (61.40%)	0.24 (0.12-4.83)	0.4579

After performing gene polymorphism of the NAT2 gene, the patients were divided into rapid and slow acetylators on the basis of the presence or absence of mutant alleles. The presence of any two mutant alleles for NAT2 variants (NAT2*5, NAT2*6, or NAT2*7) was defined as a slow acetylator profile, whereas a rapid acetylator presents one (intermediate) or two (rapid) wild-type NAT2*4 alleles. The frequency of a fast-acetylator variant NAT2*4/*4 of the NAT2 genotype was 0% in the ATDIH group whereas it was 100% in the tolerant control group. The frequency of a slow acetylator variant NAT2*6/7 of the NAT2 genotype was 15.78% and 9.3% in the ATDIH group and the tolerant control group, respectively. There was no significant difference between the ATDIH group and the tolerant control group (OR: 0.06; CI: 0.01-4.79, p=0.458). The NAT2*5/7 slow acetylator of the NAT2 genotype was 73.68% and 72.09% in the ATDIH group and the tolerant control group, respectively. There was no significant difference between the ATDIH group and the tolerant control group (OR: 0.24; CI: 0.00-2.33, p=0.277), respectively. The NAT2*5/6 slow acetylator of the NAT2 genotype was 10.52% and 18.66% in the ATDIH group and the tolerant control group, respectively. There was no significant difference between the ATDIH group and the tolerant control group (OR: 0.37; CI: 0.01-9.69, p=0.904). The frequencies of intermediate acetylators NAT2*5/4, NAT2*6/4, and NAT2*7/4 were 18.18%, 36.36%, and 45.45% in the ATDIH group whereas they were 4.34%, 65.21%, and 30.43% in the tolerant control group, respectively. The frequencies of intermediate acetylators NAT2*5/4, NAT2*6/4, and NAT2*7/4 were comparable between the TDIH and tolerant control groups (p>0.05) (Table 4).

**Table 4:** Genotypes frequencies of N-acetyltransferase-2 (NAT2) in the ATDIH and tolerant control groups Data are represented as n (%). ATDIH: antituberculosis drug-induced hepatotoxicity; OR: odds ratio; CI: confidence intervals

	ATDIH Group (n=30)	Tolerant Control Group (n=70)	OR (CI)	p-value
Fast acetylators	n=0	n=4		
NAT2*4/4	0 (0%)	4 (100%)	Reference	-
Intermediate acetylators	n=11	n=23		
NAT2*5/4	2 (18.18%)	1 (4.34%)	0.06 (0.00-2.33)	0.2771
NAT2*6/4	4 (36.36%)	15 (65.21%)	0.38 (0.01-8.54)	0.7764
NAT2*7/4	5 (45.45%)	7 (30.43%)	0.18 (0.00-3.44)	0.3052
Slow acetylators	n=19	n=43		
NAT2*6/7	3 (15.78%)	4 (9.30%)	0.14 (0.00-3.64)	0.4056
NAT2*5/7	14 (73.68%)	31 (72.09%)	0.24 (0.01-4.79)	0.4578
NAT2*5/6	2 (10.52%)	8 (18.66%)	0.37 (0.01-9.69)	0.9039

The allele frequencies of NAT2*4, NAT2*5, NAT2*6, and NAT2*7 in the ATDIH group were 18.33%, 30.0%, 15.0%, and 36.66%, and in the tolerant control group, they were 22.14%, 28.57%, 19.28%, and 30%, respectively. The allele frequencies of NAT2*4, NAT2*5, NAT2*6, and NAT2*7 were not significantly different in the ATDIH and tolerant control groups (Table 5).

**Table 5:** Allele frequencies of N-acetyltransferase-2 (NAT2) in the hepatotoxicity and non-hepatotoxicity groups Data are represented as n (%). ATDIH: antituberculosis drug-induced hepatotoxicity; OR: odds ratio; CI: confidence intervals

	ATDIH Group (n=60)	Tolerant Control Group (n=140)	OR (CI)	p-value
NAT2*4	11 (18.33%)	31 (22.14%)	Reference	------
NAT2*5	18 (30.0%)	40 (28.57%)	0.78 (0.31-1.91)	0.7614
NAT2*6	9 (15.0%)	27 (19.28%)	1.06 (0.38-2.95)	0.9045
NAT2*7	22 (36.66%)	42 (30%)	0.67 (0.28-1.60)	0.4992

## Discussion

Tuberculosis (TB) remains an important cause of mortality and morbidity worldwide. There are more than 20 drugs available for TB treatment and they are used in various combinations, in different circumstances. More than 90% of people with drug-susceptible TB can be cured in six months using a combination of first-line TB drugs. The major side effects of anti-TB drugs that have a clinical implication include hepatotoxicity, ocular toxicity, and skin hypersensitivity reactions. Hepatotoxicity is the most serious adverse drug reaction (ADR) of anti-TB drugs, which may be due to the synergistic effect of H, R, and Z given concurrently [1-9]. The factors held responsible for hepatotoxicity include the acetylator phenotype, doses of anti-TB drugs, nutritional status of the patient, and severity of the disease. The relationship between genetic factors and antitubercular drugs-induced hepatotoxicity has been approved in previous studies [6,12-17].

In the present study, we observed that gender did not a significant difference between the hepatotoxicity (ATDIH) and tolerant control groups in eastern Uttar Pradesh patients. The findings of Bose et al. (2011), Rana et al. (2014), and Teixeira et al. (2011) support our study, who observed that age and gender were not significant risk factors for hepatotoxicity in TB patients [13-15]. Contrary to our findings, the previous study done by An et al. (2012) in the Chinese population reported that men formed a significantly higher proportion in the non-hepatotoxicity group as compared with the hepatotoxicity group [16]. The difference in these results may be due to different ethnicity. Another study also suggests that female gender with SA status is an important predictor factor for anti-TB DIH [17].

In our study, the mean age of hepatotoxicity is 32.20±7.41 years, which favors the argument that the disease is common in the economically productive age group as compared to other age groups. However, findings by other studies done by Gupta et al. (2013) in the Western India population in terms of age "median (interquartile range, IQR)" was 37 (25-49) years [18]. Another study on the North Indian population done by Rana et al. (2014) who reported that the mean age of hepatotoxicity was 43.6±18.7 years [14]. Some other studies by Bose et al. (2010) and Singla et al. (2014) reported that the mean age of hepatotoxicity was 48.17±17 years and 38±6.62 years, respectively [13,19]. Our study showed that the incidence of hepatotoxicity in the Eastern Uttar Pradesh population is more in the earlier age group. This may be due to the poor nutritional condition of a lower socioeconomic state population, which may result in a more compromised state of the liver and more prone to free radical injury.

In this study, the localization of disease or extrapulmonary organ involvement has no significant association with the development of hepatotoxicity in our study. Another study by Wang et al. in 2011 also suggested that there was no significant association between the localization of tuberculosis (pulmonary/extrapulmonary) and ATDIH [20]. However extrapulmonary organ involvement was reported to be associated with drug-induced hepatotoxicity by the studies done by Parthsarathy et al. (1986) and Sharma et al. (2002) [21-22]. This difference may be attributed to the fact that extrapulmonary tuberculosis may not necessarily indicate the severity of the disease.

In the present study, hepatotoxicity was more common in smokers (30%) as compared to the tolerant control group (8.57%), but there is no significant association. Zaverucha-do-Valle et al. (2014) observed a decreased risk of developing anti-TB DIH in active smokers when compared to non-smokers (OR: 0.28, 95 CI: 0.11-0.64; p < 0.01) in Brazil [23].

In our study, 4% of the people are fast acetylators, 34% are intermediate acetylators, and 62% are slow acetylators. There is a high percentage of slow acetylators among the Eastern Uttar Pradesh population, which is similar to the South Indian population where it was reported to be 60% by Gurumurthy et al. (1984) [24]. In slow acetylators (SA), phenotypes acetyl-hydrazine and hydrazine are the toxic metabolites of isonicotinylhydrazide (INH), which may cause liver damage. We have determined the frequency of the slow acetylator genotypes, which is higher in cases (63%) as compared to the controls (61.40%), with a p-value of 0.4579 (OR=0.24, CI= 0.12-4.83). Similarly, Gupta et al. (2013) with 185 control and 105 case groups showed that the NAT2 gene with the SA genotype was non-significant in the development of hepatotoxicity, with an OR of 1.69 (95% CI, 0.84-3.38), p=0.13 [21]. Our results are supported by similar findings of previous studies by Sharma et al. (2016), who reported that the drug-induced hepatotoxicity developed mostly in those patients who were slow acetylators (82.8%) but that the slow acetylator NAT2 genotype is not a risk factor for the development of drug-induced hepatotoxicity [22]. Bose et al. (2011) reported a significantly higher frequency of slow-acetylator genotypes in DIH patients (70.73%) when compared to non-DIH patients (44.63%), with OR 2.99 (95% CI, 1.4-6.2), P=0.0045 [13]. Roy et al. (2006) compared the NAT2 gene polymorphism in 33 patients with pulmonary TB who developed drug-induced hepatotoxicity and 33 TB patients who did not develop drug-induced hepatotoxicity [25]. Their study also did not reveal significant differences in the NAT2 slow-acetylator allele frequencies between cases and controls.

In our current study, the NAT2*5 allele frequency is 30% in the hepatotoxic group and 28.57% in the non-hepatotoxic group in the Eastern Uttar Pradesh population, which is in agreement with a previous study based in India indicating that this allele is very common in the Indian/South Asian population [13]. However, these data are not concordant with the findings of another study on ATT-induced hepatitis in Taiwan by Huang et al. (2002) who mentioned that the NAT2*5 allele was rare in Asia; the NAT2*5 allele had the lowest frequency (7.14%, 16/224) here [26].

In the present study, the prevalence of the NAT2*6 allele is low, that is, 15% in the case group and 19.28% in the control group, which has no significant relationship with hepatotoxicity. A similar finding but with a higher proportion was found in the study by Gupta et al. (2013) with 36% case and 33.33% control, which has no significant association with hepatotoxicity [18]. The prevalence of the NAT2*6 allele is low in East Asians, with approximately 20% reported in Korea and Taiwan [26-28]. Nevertheless, Kim and colleagues have reported that the NAT2*6 allele was significantly associated with antituberculosis drugs-drug-induced liver injury (ATD-DILI) susceptibility (p-value=0.0016) in the Korean population [29]. Similar observations were found by Huang and colleagues, where a higher frequency of the NAT2*6 allele was detected in DILI cases as compared to the ATD-tolerant controls (p<0.05) among the Taiwanese [26].

In the present study, the prevalence of the NAT2*7 allele is higher in the Eastern Uttar Pradesh population, that is, 36.66% in the case group and 30% in the control group, which has no significant relationship with hepatotoxicity OR=0.67 (CI=0.28-1.60). Similarly, Lv et al. (2012) show that the allele frequencies of NAT2*7 of NAT 2 gene polymorphism did not differ significantly between cases and controls [29]. The NAT2*7 allele was rare among Europeans (2%) as compared to South Asians (11%) [30]. When the three alleles were further assigned into the slow and rapid acetylator phenotypes, both European and South Asian cohorts showed a similar frequency of slow acetylator distribution (50%-60%), higher than those reported in East Asian cohorts (10%) [30]. The contradictory findings of the present study with those of the other studies conducted on Asian populations could be due to the difference in the statistical analysis approach to evaluate the risk factor, sample size, and different selection criteria used for cases and controls.

Limitations

We had less than one year to complete our study with a small sample size. The incidence of antitubercular drug-induced hepatotoxicity is very low. Our study is single-centered, not multicentered. Our study is a tertiary level, hospital-based study and not a community-based or endogenous study. Due to a lack of funding, we were unable to include a measurement of the plasma concentration of drugs and its metabolites. For a better outcome, we have to study other genes and oxidizing radicals.

## Conclusions

We conclude that NAT2 gene polymorphism was not associated with drug-induced hepatotoxicity in tubercular patients. Gender, age, smoking, drug abuse, and category of treatment were also not risk factors for the development of hepatotoxicity during tuberculosis treatment. Further studies with large sample sizes will be necessary to confirm our findings.
